# Genome-wide association analysis of hippocampal volume identifies enrichment of neurogenesis-related pathways

**DOI:** 10.1038/s41598-019-50507-3

**Published:** 2019-10-10

**Authors:** Emrin Horgusluoglu-Moloch, Shannon L. Risacher, Paul K. Crane, Derrek Hibar, Paul M. Thompson, Andrew J. Saykin, Kwangsik Nho, Alzheimer’s Disease Neuroimaging Initiative (ADNI), Michael W. Weiner, Paul Aisen, Ronald Petersen, Clifford R. Jack, William Jagust, John Q. Trojanowki, Arthur W. Toga, Laurel Beckett, Robert C. Green, John Morris, Leslie M. Shaw, Jeffrey Kaye, Joseph Quinn, Lisa Silbert, Betty Lind, Raina Carter, Sara Dolen, Lon S. Schneider, Sonia Pawluczyk, Mauricio Beccera, Liberty Teodoro, Bryan M. Spann, James Brewer, Helen Vanderswag, Adam Fleisher, Judith L. Heidebrink, Joanne L. Lord, Sara S. Mason, Colleen S. Albers, David Knopman, Kris Johnson, Rachelle S. Doody, Javier Villanueva-Meyer, Munir Chowdhury, Susan Rountree, Mimi Dang, Yaakov Stern, Lawrence S. Honig, Karen L. Bell, Beau Ances, John C. Morris, Maria Carroll, Mary L. Creech, Erin Franklin, Mark A. Mintun, Stacy Schneider, Angela Oliver, Daniel Marson, Randall Griffith, David Clark, David Geldmacher, John Brockington, Erik Roberson, Marissa Natelson Love, Hillel Grossman, Effie Mitsis, Raj C. Shah, Leyla deToledo-Morrell, Ranjan Duara, Daniel Varon, Maria T. Greig, Peggy Roberts, Marilyn Albert, Chiadi Onyike, Daniel D’Agostino, Stephanie Kielb, James E. Galvin, Brittany Cerbone, Christina A. Michel, Dana M. Pogorelec, Henry Rusinek, Mony J de Leon, Lidia Glodzik, Susan De Santi, P. Murali Doraiswamy, Jeffrey R. Petrella, Salvador Borges-Neto, Terence Z. Wong, Edward Coleman, Charles D. Smith, Greg Jicha, Peter Hardy, Partha Sinha, Elizabeth Oates, Gary Conrad, Anton P. Porsteinsson, Bonnie S. Goldstein, Kim Martin, Kelly M. Makino, M. Saleem Ismail, Connie Brand, Ruth A. Mulnard, Gaby Thai, Catherine Mc-Adams-Ortiz, Kyle Womack, Dana Mathews, Mary Quiceno, Allan I. Levey, James J. Lah, Janet S. Cellar, Jeffrey M. Burns, Russell H. Swerdlow, William M. Brooks, Liana Apostolova, Kathleen Tingus, Ellen Woo, Daniel H. S. Silverman, Po H. Lu, George Bartzokis, Neill R Graff-Radford, Francine Parfitt, Tracy Kendall, Heather Johnson, Martin R. Farlow, Ann Marie Hake, Brandy R. Matthews, Jared R. Brosch, Scott Herring, Cynthia Hunt, Christopher H. van Dyck, Richard E. Carson, Martha G. MacAvoy, Pradeep Varma, Howard Chertkow, Howard Bergman, Chris Hosein, Sandra Black, Bojana Stefanovic, Curtis Caldwell, Ging-Yuek Robin Hsiung, Howard Feldman, Benita Mudge, Michele Assaly, Elizabeth Finger, Stephen Pasternack, Irina Rachisky, Dick Trost, Andrew Kertesz, Charles Bernick, Donna Munic, MarekMarsel Mesulam, Kristine Lipowski, Sandra Weintraub, Borna Bonakdarpour, Diana Kerwin, Chuang-Kuo Wu, Nancy Johnson, Carl Sadowsky, Teresa Villena, Raymond Scott Turner, Kathleen Johnson, Brigid Reynolds, Reisa A. Sperling, Keith A. Johnson, Gad Marshall, Jerome Yesavage, Joy L. Taylor, Barton Lane, Allyson Rosen, Jared Tinklenberg, Marwan N. Sabbagh, Christine M. Belden, Sandra A. Jacobson, Sherye A. Sirrel, Neil Kowall, Ronald Killiany, Andrew E. Budson, Alexander Norbash, Patricia Lynn Johnson, Thomas O. Obisesan, Saba Wolday, Joanne Allard, Alan Lerner, Paula Ogrocki, Curtis Tatsuoka, Parianne Fatica, Evan Fletcher, Pauline Maillard, John Olichney, Charles DeCarli, Owen Carmichael, Smita Kittur, Michael Borrie, T-Y Lee, Rob Bartha, Sterling Johnson, Sanjay Asthana, Cynthia M. Carlsson, Steven G. Potkin, Adrian Preda, Dana Nguyen, Pierre Tariot, Anna Burke, Nadira Trncic, Adam Fleisher, Stephanie Reeder, Vernice Bates, Horacio Capote, Michelle Rainka, Douglas W. Scharre, Maria Kataki, Anahita Adeli, Earl A. Zimmerman, Dzintra Celmins, Alice D. Brown, Godfrey D. Pearlson, Karen Blank, Karen Anderson, Laura A. Flashman, Marc Seltzer, Mary L. Hynes, Robert B. Santulli, Kaycee M. Sink, Leslie Gordineer, Jeff D. Williamson, Pradeep Garg, Franklin Watkins, Brian R. Ott, Henry Querfurth, Geoffrey Tremont, Stephen Salloway, Paul Malloy, Stephen Correia, Howard J. Rosen, Bruce L. Miller, David Perry, Jacobo Mintzer, Kenneth Spicer, David Bachman, Nunzio Pomara, Raymundo Hernando, Antero Sarrael, Norman Relkin, Gloria Chaing, Michael Lin, Lisa Ravdin, Amanda Smith, Balebail Ashok Raj, Kristin Fargher

**Affiliations:** 1 Department of Medical and Molecular Genetics, Indiana University School of Medicine, Indianapolis, IN USA; 2 Present Address: Department of Genetics and Genomic Sciences, Icahn Institute of Genomics and Multiscale Biology, Icahn School of Medicine at Mount Sinai, New York, NY USA; 3 Center for Neuroimaging, Department of Radiology and Imaging Sciences, Indiana University School of Medicine, Indianapolis, IN USA; 4 Indiana Alzheimer Disease Center, Indiana University School of Medicine, Indianapolis, IN USA; 5 Department of Medicine, University of Washington, School of Medicine, Seattle, WA USA; 6 Imaging Genetics Center, Mark and Mary Stevens Neuroimaging and Informatics Institute, USC Keck School of Medicine, University of Southern California, Los Angeles, CA USA; 7 Neuroscience Biomarkers, Janssen Research and Development, LLC, San Diego, CA USA; 8 Center for Computational Biology and Bioinformatics, Indiana University School of Medicine, Indianapolis, IN USA; 9 Magnetic Resonance Unit at the VA Medical Center and Radiology, Medicine, Psychiatry and Neurology, University of California, San Francisco, USA; 10 San Diego School of Medicine, University of California, California, USA; 11 Mayo Clinic, Minnesota, USA; 12 Mayo Clinic, Rochester, USA; 13 University of California, Berkeley, USA; 14 University of Pennsylvania, Pennsylvania, USA; 15 University of Southern California, California, USA; 16 University of California, Davis, California, USA; 17 MPH Brigham and Women’s Hospital/Harvard Medical School, Massachusetts, USA; 18 Indiana University, Indiana, USA; 19 Washington University St. Louis, Missouri, USA; 20 Oregon Health and Science University, Oregon, USA; 21 University of California–San Diego, California, USA; 22 University of Michigan, Michigan, USA; 23 Baylor College of Medicine, Houston, State of Texas USA; 24 Columbia University Medical Center, South Carolina, USA; 25 University of Alabama –, Birmingham, Alabama USA; 26 Mount Sinai School of Medicine, New York, USA; 27 Rush University Medical Center, Rush University, Illinois, USA; 28 Wien Center, Florida, USA; 29 Johns Hopkins University, Maryland, USA; 30 New York University, New York, NY USA; 31 Duke University Medical Center, North Carolina, USA; 32 University of Kentucky, Kentucky, USA; 33 University of Rochester Medical Center, New York, NY USA; 34 University of California, Irvine, California, USA; 35 University of Texas Southwestern Medical School, Texas, USA; 36 Emory University, Georgia, USA; 37 University of Kansas, Medical Center, Kansas, USA; 38 University of California, Los Angeles, California, USA; 39 Mayo Clinic, Jacksonville, USA; 40 Yale University School of Medicine, Connecticut, USA; 41 McGill University, Montreal-Jewish General Hospital, Montreal, Canada; 42 Sunnybrook Health Sciences, Ontario, USA; 43 U.B.C. Clinic for AD & Related Disorders, Vancouver, Canada; 44 Cognitive Neurology - St. Joseph’s, Ontario, USA; 45 Cleveland Clinic Lou Ruvo Center for Brain Health, Ohio, USA; 46 Northwestern University, Evanston, USA; 47 Premiere Research Inst (Palm Beach Neurology), West Palm Beach, USA; 48 Georgetown University Medical Center, Washington D.C, USA; 49 Brigham and Women’s Hospital, Massachusetts, USA; 50 Stanford University, California, USA; 51 Banner Sun Health Research Institute, Sun City, USA; 52 Boston University, Massachusetts, USA; 53 Howard University, Washington D.C, USA; 54 Case Western Reserve University, Ohio, USA; 55 University of California, Davis – Sacramento, California, USA; 56 Neurological Care of CNY, New York, USA; 57 Parkwood Hospital, Pennsylvania, USA; 58 University of Wisconsin, Wisconsin, USA; 59 University of California, Irvine, BIC USA; 60 Banner Alzheimer’s Institute, Phoenix, USA; 61 Dent Neurologic Institute, New York, NY USA; 62 Ohio State University, Ohio, USA; 63 Albany Medical College, New York, NY USA; 64 Hartford Hospital, Olin Neuropsychiatry Research Center, Connecticut, USA; 65 Dartmouth-Hitchcock Medical Center, New Hampshire, USA; 66 Wake Forest University Health Sciences, North Carolina, USA; 67 Rhode Island Hospital, state of Rhode Island, Providence, USA; 68 Butler Hospital, Providence, Rhode Island USA; 69 University of California, San Francisco, USA; 70 Medical University South Carolina, Charleston, USA; 71 Nathan Kline Institute, Orangeburg, New York, USA; 72 Cornell University, Ithaca, New York, USA; 73 USF Health Byrd Alzheimer’s Institute, University of South Florida, Tampa, USA

**Keywords:** Genome-wide association studies, Adult neurogenesis

## Abstract

Adult neurogenesis occurs in the dentate gyrus of the hippocampus during adulthood and contributes to sustaining the hippocampal formation. To investigate whether neurogenesis-related pathways are associated with hippocampal volume, we performed gene-set enrichment analysis using summary statistics from a large-scale genome-wide association study (N = 13,163) of hippocampal volume from the Enhancing Neuro Imaging Genetics through Meta-Analysis (ENIGMA) Consortium and two year hippocampal volume changes from baseline in cognitively normal individuals from Alzheimer’s Disease Neuroimaging Initiative Cohort (ADNI). Gene-set enrichment analysis of hippocampal volume identified 44 significantly enriched biological pathways (FDR corrected *p*-value < 0.05), of which 38 pathways were related to neurogenesis-related processes including neurogenesis, generation of new neurons, neuronal development, and neuronal migration and differentiation. For genes highly represented in the significantly enriched neurogenesis-related pathways, gene-based association analysis identified *TESC*, *ACVR1*, *MSRB3*, and *DPP4* as significantly associated with hippocampal volume. Furthermore, co-expression network-based functional analysis of gene expression data in the hippocampal subfields, CA1 and CA3, from 32 normal controls showed that distinct co-expression modules were mostly enriched in neurogenesis related pathways. Our results suggest that neurogenesis-related pathways may be enriched for hippocampal volume and that hippocampal volume may serve as a potential phenotype for the investigation of human adult neurogenesis.

## Introduction

Neurons are generated from neural stem cells in two regions of the brain, the dentate gyrus of the hippocampus and the olfactory bulb throughout the life span. Dentate gyrus (DG) neurons are incorporated into the hippocampal network. Adult neurogenesis-related pathways include signaling transduction, epigenetic regulation, immune system, proliferation of progenitor cells and differentiation, migration, and maturation of adult neurons^1–3^. Adult neurogenesis in DG of the hippocampus is regulated by multiple intrinsic and extrinsic factors such as hormones, transcription factors, cell cycle regulators and environmental factors that control neural stem cell (NSC) proliferation, maintenance, and differentiation into mature neurons. The estimated annualized hippocampal atrophy rate is 1.41% for cognitively normal older adults and in adults, new neurons are added in each hippocampus daily via adult neurogenesis with an annual turnover of 1.75% and a modest decline during aging^4,5^. Combination of structural MRI and immunohistological markers for newborn neurons and neural stem/progenitor cells in neurogenesis-related brain regions in mice revealed that neurogenesis is associated with increased hippocampal gray matter volumes in mice^6,7^. There is hippocampal atrophy and reduction of hippocampal neurogenesis in adult rats exposed to oxygen deprivation during birth^8^. Recently, it has been found that cognitively normal individuals had preserved neurogenesis compared to less angiogenesis and neuroplasticity^9^. Environmental factors enhance transcriptional and epigenetic changes between ventral and dorsal part of the dentate gyrus that may have an effect on hippocampal volume^10^. Molecular pathways and genes affect the induction of neurogenic niche and neural/progenitor cell turnover to newborn neurons for the formation of the hippocampal structure during hippocampal neurogenesis.

To our knowledge, there is no study assessing the association of adult neurogenesis related pathways with hippocampal volume measured from MRI scans in living people. In this study, in order to investigate whether genetic variants associated with variation in hippocampal volume are enriched for neurogenesis-related pathways, we performed a gene set enrichment analysis using summary statistics from a large-scale human neuroimaging genetics meta-analysis from the Enhancing Neuro Imaging Genetics through Meta-Analysis (ENIGMA) Consortium (N~13,000). Neurogenesis is an important contributor to the formation of the hippocampus in mice but less is known about the relationship between human adult neurogenesis and hippocampal volume/atrophy.

## Materials and Method

### Enhancing neuro imaging genetics through meta-analysis (ENIGMA)

The Enhancing Neuro Imaging Genetics through Meta-Analysis (ENIGMA) Consortium was initiated in December 2009. The research group involved in neuroimaging and genetics worked together on a range of large-scale studies that integrated data from 70 institutions worldwide. The goal of ENIGMA was to merge neuroimaging data with genomic data to identify common genetic variants that might affect brain structure. The first project of ENIGMA focused on identifying common genetic variants associated with hippocampal volume or intracranial volume (ICV)^11^. The aim of ENIGMA2, follow-on study of ENIGMA1, was to perform genome-wide association study (GWAS) using subcortical volumes as phenotypes^12^. In ENIGMA2, GWAS was conducted using mean hippocampal volume as a phenotype controlling for age, age^2^, sex, ancestry (the first four multidimensional scaling components), ICV, and diagnostic status, and MRI scanner (when multiple scanners were used at the same site), and genetic imputation were processed and examined by following standardized protocols freely available online (http://enigma.ini.usc.edu/protocols/imaging-protocols/). In this study, we used GWAS summary statistics in the discovery sample of 13,163 subjects of European ancestry from the ENIGMA Consortium^12^. 3,824 of the 13,163 participants (21%) have anxiety, Alzheimer’s disease, attention-deficit/hyperactivity disorder, bipolar disorder, epilepsy, major depressive disorder or schizophrenia, and the remaining 9,339 (79%) are cognitively normal subjects.

### Alzheimer’s disease neuroimaging initiative (ADNI)

The Alzheimer’s Disease Neuroimaging Initiative (ADNI) was launched in 2003 by the National Institute on Aging, the National Institute of Biomedical Imaging and Bioengineering, the Food and Drug Administration (FDA), private pharmaceutical companies, and nonprofit organizations as a public-private partnership, led by Principal Investigator Michael W. Weiner, MD, and recruited from 59 sites across the U.S. and Canada. ADNI includes over 1700 subjects consisting of cognitively normal older individuals (CN), significant memory concern (SMC), mild cognitive impairment (MCI) and Alzheimer’s Disease (AD) aged 55–90 (http://www.adni-info.org/). The primary goal of ADNI has been to test whether serial magnetic resonance imaging (MRI), positron emission tomography (PET), other biological markers, and clinical and neuropsychological assessment can be combined to measure the progression of MCI and early AD. Participants for this study included 367 CN, 94 SMC, 280 early MCI, 512 late MCI and 310 AD. Demographic information, APOE, clinical information, neuroimaging and GWAS genotyping data were downloaded from the ADNI data repository (http://adni.loni.usc.edu). The CN group does not have any significant memory concern or impairment of their daily activities. The SMC group has self-reported significant memory concerns quantified using the Cognitive Change Index^13^ and the Clinical Dementia Rating (CDR) of zero. Individuals with MCI and AD have to have memory complains. The range of Mini-Mental State Examination (MMSE) score was 24–30 for CN and MCI, and 20–26 for AD as well as objective memory loss measured by education-adjusted scores on Wechsler Memory Scale-Revised (WMS-R) Logical Memory II^14^. As diagnosis criteria, CDR score was used as 0 for CN, 0.5 for MCI with the memory box score being 0.5 or greater, and 0.5–1 for AD^15^. A composite memory score was calculated using Logical Memory and the Rey Auditory Verbal Learning Test (RAVLT), as well as memory items from the AD Assessment Scale - Cognitive (ADAS-Cog) and Mini-Mental State Examination (MMSE)^16^. Hippocampal volume was determined using MRI scans and FreeSurfer version 5.1 was used to extract hippocampal and total intracranial volumes (ICV)^17–20^. Table 1 shows selected demographic and clinical characteristics of these participants at baseline.

**Table 1:** Demographic and clinical characteristics of ADNI participants.

	CN	SMC	EMCI	LMCI	AD
N	367	94	280	512	310
Age (SD)	74.59 (5.57)	71.77 (5.65)	71.14 (7.26)	73.52 (7.65)	74.65 (7.79)
Sex (M/F)	192/175	38/56	158/122	318/194	176/134
Education (SD)	16.32 (2.68)	16.81 (2.57)	16.08 (2.67)	15.97 (2.91)	15.23 (2.97)
APOE (ε4−/ε4+)	267/99	62/32	160/119	232/280	104/206
MMSE (SD)	29.07 (1.11)	29.06 (1.16)	28.34 (1.56)	27.24 (1.79)	23.26 (2.04)
Composite score for memory (SD)	0.93 (0.532)	0.94 (0.46)	0.52 (0.49)	−0.04 (0.58)	−0.77 (0.53)
Intracranial volume (SD)	1523924 (155259)	1466989 (150559)	1513733 (151765)	1560894 (167738)	1535767 (180536)
Hippocampal volume (SD)	3612.7 (463)	3796 (471)	3633.5 (510)	3163.3 (564)	2840.4 (509)

### Genotyping data and quality control

The genotyping data of ADNI participants were collected using the Illumina Human 610-Quad, HumanOmni Express, and HumanOmni 2.5 M BeadChips. Standard quality control procedures of GWAS data for genetic markers and subjects were performed using PLINK v1.07 (pngu.mgh.harvard.edu/∼purcell/plink). Quality control procedures included excluding samples and SNPs with criteria including SNP call rate < 95%, Hardy-Weinberg equilibrium test *p* < 1 × 10^−6^, and frequency filtering (MAF < 5%), participant call rate < 95%, sex check and identity check for related individuals^21–25^. Non-Hispanic Caucasian participants were selected using HapMap 3 genotype data and the multidimensional scaling (MDS) analysis (Supplementary Fig. 1) after performing standard quality control procedures for genetic markers and subjects. For imputation of un-genotyped SNPs, MaCH (Markov Chain Haplotyping) software based on the 1000 Genomes Project as a reference panel was used^26,27^.

### Gene-set enrichment analysis

Gene-set enrichment analysis using GWAS summary statistics was performed to identify pathways and functional gene sets with significant associations with hippocampal volume. All SNPs (n = 6,571,356) and subjects with European ancestry were included in this study. Pathway annotations were downloaded from the Molecular Signatures Database version 5.0 (http://www.broadinstitute.org/gsea/msigdb/index.jsp/). This annotation data comprised a collection of Gene Ontology (GO). GO includes 1,454 pathways and is publicly available. 825 gene sets are assigned to GO biological processes, 233 gene sets are assigned to GO cellular components, and 396 gene sets are assigned to GO molecular functions. GSA-SNP software^28^ uses a p-value of each SNP from GWAS summary statistics to test if a pathway-phenotype association is significantly different from all other pathway-phenotype associations. In GSA-SNP, all SNPs within each gene are considered in turn and the negative log of the p value is noted; all of these are ranked. To avoid spurious predictions, we used the SNP with the second highest negative log p value to summarize strength of association with each gene. Each pathway (gene set) was assessed by z-statistics for the identification of the enriched pathways^29^. Gene-set enrichment analysis was restricted to pathways containing between 10 and 200 genes. False discovery rate (FDR) with the Benjamini-Hochberg procedure was used for multiple comparison correction^30^. We identified as significantly enriched pathways with hippocampal volume with FDR-corrected *p-*value < 0.05.

### Genetic association analysis

Genome-wide gene-based association analysis using GWAS p-values was performed using KGG (Knowledge-based mining system for Genome-wide Genetic studies) software. KGG uses HYST (hybrid set-based test) to determine the overall association significance in a set of SNPs at the gene level. HYST is the combination of the gene-based association test using extended Simes procedure (GATES) and the scaled chi-square test^31,32^. First, SNPs in each gene were divided into different LD blocks depending on pairwise LD coefficients (r^2^) for all SNPs. Second, for each block, a block-based p-value for association was calculated, and the key SNP was derived and marked. Next, the block-based p-values were combined accounting for LD between the key SNPs using the scaled chi-square^33^.

Targeted gene-based association analysis was performed using a set-based test in Plink v1.07 (http://pngu.mgh.harvard.edu/purcell/plink/)^22^. SNPs with *p* < 0.05 for each gene were chosen. A mean test statistic for each SNP within a gene was computed to determine with which other SNPs it is in linkage disequilibrium (LD); i.e., if the correlation coefficient between them was r^2^ > 0.5. A quantitative trait analysis (QT) was then performed with each SNP. For each gene, the top independent SNPs (i.e., not in LD; maximum of 5) are selected if their p-values are less than 0.05. The SNP with the smallest *p*-value is selected first; subsequent independent SNPs are selected in order of decreasing statistical significance. From these subsets of SNPs, the statistic for each gene is calculated as the mean of these single SNP statistics^34^. The analysis was performed using an additive model or in other words, the additive effect of the minor allele on the phenotypic mean was estimated^22,35^. Covariates included age, sex, years of education, and diagnosis for composite scores for memory. An empirical *p*-value (20,000 permutations) was reported for each gene for multiple comparison adjustment^22^.

### Gene expression correlation analysis

We analyzed gene expression data in the hippocampal subfields, CA1 and CA3, from 32 normal controls brain samples in the Gene Expression Omnibus (GEO) repository at the National Center for Biotechnology Information (NCBI) archives. The Illumina HumanHT-12 v3 Expression BeadChip (48,803 probes) was used to measure expression of over 25,000 annotated genes. We processed gene expression data and removed the outliers as previously described^36^. We excluded probes if they were present in three or fewer samples or if they do not correspond to any gene symbol annotations. Lastly we removed duplicate probes for a gene and kept only the probe with the highest expression level. After all data cleaning process, 15,037 genes remained. We performed a weighted gene correlation network analysis (WGCNA) using processed expression data to identify clusters of highly correlated genes expressed in specific brain regions (CA1 and CA3) as modules. Pearson correlations between gene pairs were calculated. This matrix was transformed into a signed adjacency matrix by using a power function. Then, topological overlap (TO) was calculated by using the components of this matrix. Genes were clustered hierarchically by the distance measure, 1-TO, and the dynamic tree algorithm determined initial module assignments^37^. Gene module membership between each gene and each module eigengene was calculated. We tested these modules for enrichment of neurogenesis-related pathways.

## Results

**Figure 1:**
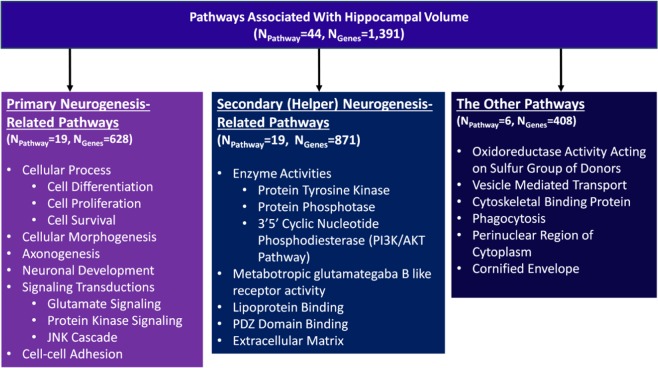
Conceptual classification of 44 pathways significantly enriched for hippocampal volume.

Gene-set enrichment analysis using large-scale GWAS summary statistics for hippocampal volume (N = 13,163) identified 44 significantly enriched biological pathways (FDR-corrected *p*-value < 0.05) (Table 2) including 38 pathways related to neurogenesis (Supplementary Table S1). We classified the 38 neurogenesis-related pathways as primary (N = 19) and secondary (helper) (N = 19) based on existing knowledge and literature mining (Fig. 1). The primary neurogenesis-related pathways were related to cellular processes such as neuronal proliferation, differentiation and survival, cellular morphogenesis, axonogenesis, neuronal development, signal transduction, and cell-cell adhesion. The secondary neurogenesis-related pathways consisted of enzyme activities related to neurogenesis, metabotropic receptor activity, lipoprotein binding and extracellular matrix. Six pathways were not related to any neurogenesis-related process such as oxidoreductase activity, phagocytosis, perinuclear region of cytoplasm and cornified envelope.

**Table 2:** Molecular Signatures Database (MSigDB) GO Ontology pathways enriched for hippocampal volume.

Pathways	# of genes/set size	Corrected *p*-value
Oxidoreductase Activity Acting On Sulfur Group Of Donors	10/10	4.68 × 10^−4^
Neuron differentiation	73/76	0.001181
Cell Projection	105/108	0.001181
Microvillus	11/11	0.001479
Neurite Development	51/53	0.00312
Cell Recognition	18/19	0.00312
Generation of Neurons	80/83	0.00312
Transmembrane Receptor Protein Kinase Activity	50/51	0.00312
Protein Domain Specific Binding	71/72	0.00312
Neuron Development	59/61	0.003242
Axonogenesis	41/43	0.003242
Cellular Morphogenesis During Differentiation	47/49	0.004265
Neurogenesis	90/93	0.005646
Transmembrane Receptor Protein Tyrosine Kinase Activity	42/43	0.005903
Vesicle Mediated Transport	188/194	0.011803
Glutamate Receptor Activity	20/20	0.011803
Cytoskeletal Protein Binding	153/159	0.011803
Jnk Cascade	45/47	0.011925
Stress Activated Protein Kinase Signaling Pathway	47/49	0.013007
Metabotropic Glutamategaba B Like Receptor Activity	10/10	0.01599
Phagocytosis	16/17	0.018307
Regulation of Axonogenesis	10/10	0.018307
Regulation of Anatomical Structure Morphogenesis	24/25	0.018307
Perinuclear Region of Cytoplasm	51/54	0.018746
Glutamate Signaling Pathway	16/17	0.021249
Cornified Envelope	12/13	0.023212
Lipoprotein Binding	18/18	0.024574
Pdz domain Binding	14/14	0.025352
Protein Tyrosine Kinase Activity	62/63	0.026949
3 5 Cyclic Nucleotide Phosphodiesterase Activity	13/13	0.026949
Negative Regulation of Cell Proliferation	148/156	0.02873
Protein Oligomerization	35/40	0.02873
Exopeptidase Activity	29/32	0.02873
Extracellular Matrix	95/100	0.030238
Cell Cell Adhesion	83/86	0.030238
Proteinaceous Extracellular Matrix	93/98	0.030238
Maintenance of Protein Localization	12/13	0.030238
Maintenance Of Cellular Protein Localization	11/11	0.030238
Transmembrane Receptor Protein Phosphatase Activity	19/19	0.030238
Cell Projection Biogenesis	23/25	0.030415
Cyclic Nucleotide Phosphodiesterase Activity	14/14	0.030799
Central Nervous System Development	110/123	0.030799
Protein Tyrosine Phosphatase Activity	52/53	0.031472
Active Transmembrane Transporter Activity	113/122	0.041004

Since the inhibition of neurogenesis could be relevant to hippocampal atrophy^38^, we also examined if neurogenesis-related pathways were enriched with hippocampal atrophy over two years from baseline in cognitively normal individuals without amyloid-β pathology based on [^18^F]Florbetapir PET or CSF amyloid-β measurement (N = 112) in ADNI. Seven pathways related to neurogenesis processes were significantly enriched with hippocampal atrophy (FDR-corrected *p*-value < 0.05) in cognitively normal adults (Supplementary Table S2). These pathways were related to cellular differentiation, cellular morphogenesis during development, neurite development, axonogenesis, cell-cell adhesion and neuron development (Table 3).

**Table 3:** Molecular Signatures Database (MSigDB) GO Ontology pathways enriched with hippocampal atrophy over 2 years from baseline.

Pathway (n = 7)	# of genes/set size	Corrected *p*-value
Cellular Morphogenesis During Differentiation	33/49	0.0082
Regulation of Anatomical Structure Morphogenesis	18/25	0.0082
Neurite Development	34/53	0.0082
Axonogenesis	30/43	0.013
Cell-Cell Adhesion	54/86	0.013
Neuron Development	40/61	0.050
Transmembrane Receptor Protein Phosphatase Activity	15/19	0.050

**Figure 2:**
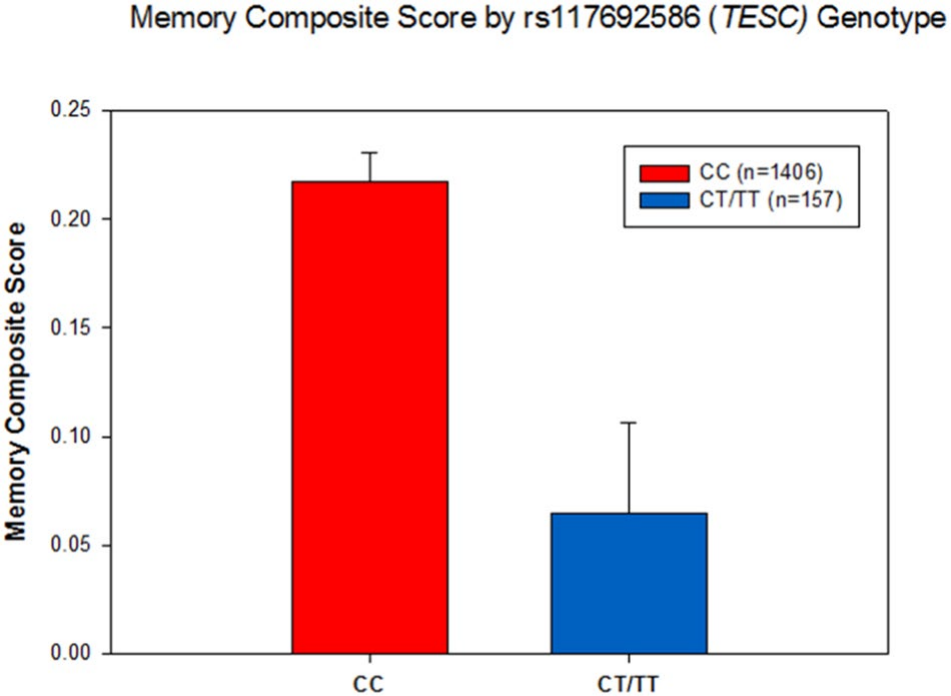
rs117692586 in *TESC* is significantly associated with composite scores for memory. Subjects with at least one copy of the minor allele (T) of rs117692586 showed poorer memory performance compared to those without the minor allele (*p*-value ≤ 0.001).

Furthermore, we performed targeted gene-based association analysis of hippocampal neurogenesis related pathway associated candidate genes using ENIGMA GWAS summary statistics^31^. The gene-based analysis revealed that 4 genes (*MSRB3*, *TESC*, *DPP4*, and *ACVR1*) were significantly associated with hippocampal volume (corrected *p*-value < 0.05; Table 4). Since hippocampal volume is correlated with memory performance, we performed an association analysis of these four genes (with 682 SNPs) with composite memory scores in ADNI. The gene-based association analysis showed that *TESC* is significantly associated with composite memory scores after adjusting for multiple testing (*p*-value* = *5.7 × 10^−3^; Table 5). One novel SNP (rs117692586) upstream of *TESC* was significantly associated with composite memory scores (*p*-value = 4.3 × 10^−4^; Table 6). rs117692586-T is associated with poorer memory performance (Fig. 2).

**Table 4:** Gene-based association analysis results (*p*-value) of four significant genes for hippocampal volume using common variants (MAF ≥ 0.05).

Gene	Corrected *p*-value
*MSRB3*	3.4 × 10^−6^
*TESC*	1.3 × 10^−2^
*DPP4*	3.7 × 10^−2^
*ACVR1*	4.8 × 10^−2^

**Table 5:** Gene-based association analysis results (*p*-values) of four genes for composite scores for memory using common variants (MAF ≥ 0.05) in ADNI, where empirical p-values were calculated using 20,000 permutations.

Gene	ADNI (N = 1,563)	
	p-value	Significant Independent SNP
*MSRB3*	0.26	rs7294862|rs6581626
***TESC***	**5**.**7** × **10**^**−3**^	**rs117692586|rs12302906**
*DPP4*	0.26	rs35635667|rs3788979
*ACVR1*	1	NA

**Table 6:** SNP-based association analysis results in *TESC* for composite scores for memory in ADNI.

rs117692586 (*TESC*)	ADNI (N = 1,563)	
	β	*p*-value
Memory Composite Score	−0.149 (−0.231, −0.066)	4.3 × 10^−4^

Finally, we analyzed gene expression data in the Gene Expression Omnibus (GEO) repository to investigate if neurogenesis-related pathways were enriched in the CA1 and CA3 regions of the hippocampus in normal controls. A weighted gene correlation network analysis yielded 20 modules of co-expressed genes. These 20 modules were tested for enrichment of neurogenesis-related pathways. Six modules were found to be significantly enriched with neurogenesis-related pathways after adjusting for multiple testing. The six significantly enriched modules are all related to neurogenesis-related pathways such as neuronal proliferation and differentiation as well as cellular process (Table 7).

**Table 7:** Weighted gene correlation network analysis (WGCNA) results of six modules represented by colors enriched with neurogenesis-related pathways after adjusting for multiple testing.

WGCNA module	Corrected *p*-value
Green	5.2 × 10^−84^
Orange	1 × 10^−21^
Black	3.8 × 10^−17^
Darkolivegreen	4.4 × 10^−11^
Bisque4	3 × 10^−7^
Lavenderblush3	7.6 × 10^−4^

## Discussion

Using large-scale GWAS summary statistics for hippocampal volume in 13,163 subjects of European ancestry from the ENIGMA Consortium, we performed gene-set enrichment analysis to identify 44 pathways with enrichment for hippocampal volume. These enriched pathways showed that genes associated with variation in hippocampal volume are related to neurogenesis and cellular processes including neuronal cell proliferation, differentiation and maturation as well as cell adhesion. In addition, co-expression network-based functional analysis of gene expression data in the hippocampal subfields, CA1 and CA3, from 32 normal controls showed that co-expression modules were mostly enriched in neurogenesis-related pathways.

The enriched pathways showed significant relationships between neurogenesis and hippocampal volume/atrophy. Since several studies showed neurogenesis occurs in the dentate gyrus of the hippocampus^4,39^, it is not surprising that hippocampal volume is significantly related to neurogenesis-related pathways. In particular, we observed significant enrichment of pathways related to cell proliferation, neuron differentiation, neuron generation, neurite development, neuronal development, cell recognition, neurogenesis and axonogenesis. The neural progenitor cells in the subgranular zone of the hippocampus differentiate and incorporate into neural network circuitry as mature neurons in the adult human brain^4^. In addition, these newly developed neurons enhance the formation of the hippocampus during neurogenesis and many genes are involved in these processes^40,41^. Moreover, our pathway enrichment analysis found that hippocampal volume is significantly related to signal transduction processes such as glutamate signaling, protein kinase signaling, and the Jun N-Terminal Kinase (JNK) cascade. Previously we identified five neurogenesis related pathways and the signal transduction pathway was one of the important pathways in adult neurogenesis processes^3^. During adult neurogenesis, functional granule cells in the dentate gyrus of the adult hippocampus release glutamate, project to target cells in the CA3 region, and receive glutamatergic and γ-aminobutyric acid (GABA)-ergic inputs to control their spiking activity in neuronal networks that support the formation of memory and learning^42,43^. Phosphoinositide 3-kinase (PI3K)/protein kinase pathways enhance neuronal differentiation and inhibit apoptosis of progenitor cells^44,45^. In addition, studies showed that JNK1 in the JNK cascade plays a role in neuronal differentiation and neuronal and axonal maturation^46–48^. Also, it has been shown that absence of JNK1 enhances hippocampal neurogenesis and reduces anxiety-related phenotypes in mouse models^46^.

Pathways related to enzyme activities such as protein tyrosine kinases, protein tyrosine phosphatases and 3’5’ cyclic nucleotide phosphodiesterases were enriched for hippocampal volume. Studies showed that three subfamilies, Tyro3, Axl and Mertk (TAM), of receptor protein tyrosine kinases play a crucial role in adult neurogenesis. TAM receptors impact proliferation and differentiation of neural stem cells to immature neurons by controlling overproduction of pro-inflammatory cytokines^49^. Protein tyrosine phosphatases control neural stem cell differentiation during neurogenesis^50^.

Our results revealed the influence of neurogenesis pathway-related genetic variation on hippocampal volume. Particularly, two genes, tescalcin (*TESC*) and activin receptor 1 (*ACVR1*), were significantly associated with hippocampal volume. In addition, *TESC* was significantly associated with memory performance. Previous structural neuroimaging studies showed *TESC*-regulating polymorphisms are significantly associated with hippocampal volume and hippocampal gray matter structure^11,51^. *TESC* cooperates with the plasma membrane Na(+)/H(+) exchanger NHE1 that catalyzes electroneutral influx of extracellular Na(+) and efflux of intracellular H(+) and establishes intracellular pH level as well as cellular hemostasis^52,53^. *TESC* was expressed in tissues such as heart and brain and plays an important role during embryonic development^53^. *TESC* plays a crucial role in controlling cell proliferation and differentiation for the formation of the hippocampal structure during brain development^51^. In addition, *ACVR1*, a member of a protein family called bone morphogenetic protein (BMP) type I receptors, regulates the hippocampal dentate gyrus stem cells during neurogenesis^54^. In addition, our gene co-expression analysis showed that *TESC* and *ACVR1* were co-expressed together in the neurogenesis pathway-related module.

A limitation of the present report is that we used Gene Ontology pathways from MSigDB. For a pathway enrichment analysis design, there is no gold standard. There are several tools and strategies for pathway enrichment analysis, and alternate databases and algorithms for pathway enrichment analysis can affect the analytic results^55,56^. Another limitation is the lack of replication in the gene-set enrichment analysis, even though we used a large-scale GWAS result (N = 13,163). Replication in independent samples will be important. It is noteworthy that recently, Sorrell *et al*. reported that human hippocampal neurogenesis drops sharply in childhood to undetectable levels in adults, although some aspects are still under controversy^57,58^, but Boldrini *et al*. reported that healthy older adults display preserved neurogenesis^9^.

In summary, our results suggest that neurogenesis-related pathways may be enriched for hippocampal volume and that hippocampal volume may serve as a potential phenotype for the investigation of human adult neurogenesis. Genetic variation in neurogenesis pathway-related genes may have compensatory advantages or confer vulnerability to biological processes during adult neurogenesis but studies are needed to identify mechanisms by which genetic variants affect neural stem cells differentiation, proliferation, and their maturation to new neurons in human brain.

## Supplementary information

Dataset 1

## Data Availability

The data analyzed in the study are available from the ADNI website (http://adni.loni.usc.edu/) and the ENIGMA website (http://enigma.ini.usc.edu/).

## References

[1] Suh H, Deng W, Gage FH (2009). Signaling in adult neurogenesis. Annual review of cell and developmental biology.

[2] Zhao C, Deng W, Gage FH (2008). Mechanisms and functional implications of adult neurogenesis. Cell.

[3] Horgusluoglu E, Nudelman K, Nho K, Saykin AJ (2017). Adult neurogenesis and neurodegenerative diseases: A systems biology perspective. American journal of medical genetics. Part B, Neuropsychiatric genetics: the official publication of the International Society of Psychiatric Genetics.

[4] Spalding KL (2013). Dynamics of hippocampal neurogenesis in adult humans. Cell.

[5] Risacher SL (2009). Baseline MRI predictors of conversion from MCI to probable AD in the ADNI cohort. Current Alzheimer research.

[6] Biedermann SV (2016). The hippocampus and exercise: histological correlates of MR-detected volume changes. Brain Struct Funct.

[7] Sierra A, Encinas JM, Maletic-Savatic M (2011). Adult human neurogenesis: from microscopy to magnetic resonance imaging. Frontiers in neuroscience.

[8] Takada SH (2016). Impact of neonatal anoxia on adult rat hippocampal volume, neurogenesis and behavior. Behavioural brain research.

[9] Boldrini M (2018). Human Hippocampal Neurogenesis Persists throughout Aging. Cell Stem Cell.

[10] Zhang TY (2018). Environmental enrichment increases transcriptional and epigenetic differentiation between mouse dorsal and ventral dentate gyrus. Nat Commun.

[11] Stein JL (2012). Identification of common variants associated with human hippocampal and intracranial volumes. Nature genetics.

[12] Thompson PM (2014). The ENIGMA Consortium: large-scale collaborative analyses of neuroimaging and genetic data. Brain Imaging Behav.

[13] Rattanabannakit C (2016). The Cognitive Change Index as a Measure of Self and Informant Perception of Cognitive Decline: Relation to Neuropsychological Tests. J Alzheimers Dis.

[14] Kovacevic Sanja, Rafii Michael S., Brewer James B. (2009). High-throughput, Fully Automated Volumetry for Prediction of MMSE and CDR Decline in Mild Cognitive Impairment. Alzheimer Disease & Associated Disorders.

[15] Petersen RC (2010). Alzheimer’s Disease Neuroimaging Initiative (ADNI): clinical characterization. Neurology.

[16] Crane PK (2012). Development and assessment of a composite score for memory in the Alzheimer’s Disease Neuroimaging Initiative (ADNI). Brain Imaging Behav.

[17] Risacher SL (2015). APOE effect on Alzheimer’s disease biomarkers in older adults with significant memory concern. Alzheimer’s & dementia: the journal of the Alzheimer’s Association.

[18] Risacher SL (2013). The role of apolipoprotein E (APOE) genotype in early mild cognitive impairment (E-MCI). Frontiers in aging neuroscience.

[19] Dale AM, Fischl B, Sereno MI (1999). Cortical surface-based analysis. I. Segmentation and surface reconstruction. NeuroImage.

[20] Fischl B, Sereno MI, Dale AM (1999). Cortical surface-based analysis. II: Inflation, flattening, and a surface-based coordinate system. NeuroImage.

[21] Saykin AJ (2010). Alzheimer’s Disease Neuroimaging Initiative biomarkers as quantitative phenotypes: Genetics core aims, progress, and plans. Alzheimers Dement.

[22] Purcell S (2007). PLINK: a tool set for whole-genome association and population-based linkage analyses. American journal of human genetics.

[23] Ramanan VK (2014). APOE and BCHE as modulators of cerebral amyloid deposition: a florbetapir PET genome-wide association study. Molecular psychiatry.

[24] Li J (2015). Genetic Interactions Explain Variance in Cingulate Amyloid Burden: An AV-45 PET Genome-Wide Association and Interaction Study in the ADNI Cohort. BioMed research international.

[25] Saykin AJ (2015). Genetic studies of quantitative MCI and AD phenotypes in ADNI: Progress, opportunities, and plans. Alzheimers Dement.

[26] Howie B, Fuchsberger C, Stephens M, Marchini J, Abecasis GR (2012). Fast and accurate genotype imputation in genome-wide association studies through pre-phasing. Nature genetics.

[27] Nho K (2013). Whole-exome sequencing and imaging genetics identify functional variants for rate of change in hippocampal volume in mild cognitive impairment. Molecular psychiatry.

[28] Nam D, Kim J, Kim SY, Kim S (2010). GSA-SNP: a general approach for gene set analysis of polymorphisms. Nucleic acids research.

[29] Kim SY, Volsky DJ (2005). PAGE: parametric analysis of gene set enrichment. BMC bioinformatics.

[30] Benjamini Y, Hochberg Y (1995). Controlling the False Discovery Rate: A Practical and Powerful Approach to Multiple Testing. Journal of the Royal Statistical Society. Series B (Methodological).

[31] Li MX, Gui HS, Kwan JS, Sham PC (2011). GATES: a rapid and powerful gene-based association test using extended Simes procedure. American journal of human genetics.

[32] Moskvina V (2011). Evaluation of an approximation method for assessment of overall significance of multiple-dependent tests in a genomewide association study. Genetic epidemiology.

[33] Li MX, Kwan JS, Sham PC (2012). HYST: a hybrid set-based test for genome-wide association studies, with application to protein-protein interaction-based association analysis. American journal of human genetics.

[34] Horgusluoglu-Moloch E (2017). Targeted neurogenesis pathway-based gene analysis identifies ADORA2A associated with hippocampal volume in mild cognitive impairment and Alzheimer’s disease. Neurobiol Aging.

[35] Swaminathan S (2012). Amyloid pathway-based candidate gene analysis of [(11)C]PiB-PET in the Alzheimer’s Disease Neuroimaging Initiative (ADNI) cohort. Brain imaging and behavior.

[36] Miller JA, Woltjer RL, Goodenbour JM, Horvath S, Geschwind DH (2013). Genes and pathways underlying regional and cell type changes in Alzheimer's disease. Genome medicine.

[37] Langfelder P, Horvath S (2008). WGCNA: an R package for weighted correlation network analysis. BMC bioinformatics.

[38] Sapolsky RMD (2001). antidepressants, and the shrinking hippocampus. Proceedings of the National Academy of Sciences of the United States of America.

[39] Eriksson PS (1998). Neurogenesis in the adult human hippocampus. Nature medicine.

[40] Gould E, Beylin A, Tanapat P, Reeves A, Shors TJ (1999). Learning enhances adult neurogenesis in the hippocampal formation. Nature neuroscience.

[41] Aimone JB (2014). Regulation and function of adult neurogenesis: from genes to cognition. Physiological reviews.

[42] Toni N, Schinder AF (2015). Maturation and Functional Integration of New Granule Cells into the Adult. Hippocampus. Cold Spring Harbor perspectives in biology.

[43] Williamson LL, Bilbo SD (2013). Chemokines and the hippocampus: a new perspective on hippocampal plasticity and vulnerability. Brain, behavior, and immunity.

[44] Doze VA, Perez DM (2012). G-protein-coupled receptors in adult neurogenesis. Pharmacological reviews.

[45] Toledo EM, Colombres M, Inestrosa NC (2008). Wnt signaling in neuroprotection and stem cell differentiation. Progress in neurobiology.

[46] Mohammad H (2016). JNK1 controls adult hippocampal neurogenesis and imposes cell-autonomous control of anxiety behaviour from the neurogenic niche. Molecular psychiatry.

[47] Chang L, Jones Y, Ellisman MH, Goldstein LS, Karin M (2003). JNK1 is required for maintenance of neuronal microtubules and controls phosphorylation of microtubule-associated proteins. Developmental cell.

[48] Oliva AA, Atkins CM, Copenagle L, Banker GA (2006). Activated c-Jun N-terminal kinase is required for axon formation. The Journal of neuroscience: the official journal of the Society for Neuroscience.

[49] Ji R, Meng L, Li Q, Lu Q (2015). TAM receptor deficiency affects adult hippocampal neurogenesis. Metabolic brain disease.

[50] Kim SY (2016). Profiling analysis of protein tyrosine phosphatases during neuronal differentiation. Neuroscience letters.

[51] Dannlowski U (2015). Multimodal imaging of a tescalcin (TESC)-regulating polymorphism (rs7294919)-specific effects on hippocampal gray matter structure. Molecular psychiatry.

[52] Baumgartner M, Patel H, Barber DL (2004). Na(+)/H(+) exchanger NHE1 as plasma membrane scaffold in the assembly of signaling complexes. American journal of physiology. Cell physiology.

[53] Bao Y (2009). Expression and evolutionary conservation of the tescalcin gene during development. Gene expression patterns: GEP.

[54] Choe Y, Kozlova A, Graf D, Pleasure SJ (2013). Bone morphogenic protein signaling is a major determinant of dentate development. The Journal of neuroscience: the official journal of the Society for Neuroscience.

[55] Gui H, Li M, Sham PC, Cherny SS (2011). Comparisons of seven algorithms for pathway analysis using the WTCCC Crohn’s Disease dataset. BMC research notes.

[56] Ramanan VK (2012). Genome-wide pathway analysis of memory impairment in the Alzheimer’s Disease Neuroimaging Initiative (ADNI) cohort implicates gene candidates, canonical pathways, and networks. Brain imaging and behavior.

[57] Sorrells SF (2018). Human hippocampal neurogenesis drops sharply in children to undetectable levels in adults. Nature.

[58] Horgusluoglu, E. *Neurogenesis in the adult brain*, *gene networks*, *and Alzheimer’s Disease* PhD thesis, Indiana University (2017).

