# Supplementary material for: Genome-wide association analysis of hippocampal volume identifies enrichment of neurogenesis-related pathways
Source: Sci Rep. 2019 Oct 10;9:14498. doi: 10.1038/s41598-019-50507-3 (PMC6787090; doi:10.1038/s41598-019-50507-3)

Genome-wide association analysis of hippocampal volume identifies enrichment of neurogenesis-related pathways

Emrin Horgusluoglu, Ph.D.[1,2†], Shannon L. Risacher, Ph.D.[3,4], Paul K. Crane, M.D., M.P.H.[5], Derrek Hibar, Ph.D.[6,7], Paul M. Thompson, Ph.D.[6], Andrew J. Saykin, Psy.D.[1,3,4,8*], Kwangsik Nho, Ph.D.[1,3,4,8*], for the Alzheimer's Disease Neuroimaging Initiative (ADNI)[€]

[1]*Department of Medical and Molecular Genetics, Indiana University School of Medicine, Indianapolis, IN, USA;*

[2]*Department of Genetics and Genomic Sciences, Icahn Institute of Genomics and Multiscale Biology, Icahn School of Medicine at Mount Sinai, New York, NY, USA;*

[3]*Center for Neuroimaging, Department of Radiology and Imaging Sciences, Indiana University School of Medicine, Indianapolis, IN, USA;*

[4]*Indiana Alzheimer Disease Center, Indiana University School of Medicine, Indianapolis, IN, USA*

[5]*Department of Medicine, University of Washington, School of Medicine, Seattle, WA, USA;*

[6]*Imaging Genetics Center, Mark and Mary Stevens Neuroimaging and Informatics Institute, USC Keck School of Medicine, University of Southern California, Los Angeles, CA, USA;*

[7]*Neuroscience Biomarkers, Janssen Research and Development, LLC, San Diego, CA, USA*

[8]*Center for Computational Biology and Bioinformatics, Indiana University School of Medicine, Indianapolis, IN, USA;*

*These authors jointly supervised this work
† Author's current affiliation

Table S1. Full list of Molecular Signatures Database (MSigDB) GO Ontology pathways enriched for hippocampal volume.

PATHWAYS	gene count	set size	p-value	gene symbols
OXIDOREDUCTASE ACTIVITY ACTING ON SULFUR GROUP OF DONORS	10	10	3.72E-07	MSRB3, TXNDC2, TXNRD1, GSR, QSOX1, MSRA, SUOX, MSRB2, DLD, TXNRD2
NEURON DIFFERENTIATION	73	76	1.88E-06	MDGA2, RTN4, NRP1, APOE, MAPT, SLIT1, LRRC4C, NRTN, FEZ2, PARD6B, NRXN1, CYFIP1, TGFB2, LMX1B, UNC5C, CNTN4, PCSK9, ROBO1, ROBO2, PAX2, NLGN1, NRXN3, NTNG2, FARP2, LST1, FEZ1, SEMA3B, ATP2B2, TRAPPC4, BTG4, BAIAP2, MAP1S, SPON2, LDB1, PARD3, GLI2, YWHAH, POU6F2, RND1, LAMB1, KCNIP2, GHRL, YWHAG, S100B, BAI1, DPYSL5, NTNG1, AMIGO1, SLIT2, ALS2, RTN4RL1, MDGA1, PICK1, RTN1, CDK5, BRSK2, GDNF, VWC2, CDK5R1, NRP2, DTX1, PPT1, SIAH1, AGRN, NRCAM, KLK8, POU4F1, SHH, OTX2, RTN4RL2, SEMA4F, UBB, THY1, (OPHN1), (SMARCA1), (KAL1)
CELL PROJECTION	105	108	2.14E-06	TESC, TBC1D10A, ACTN2, MAPT, ABI1, FGD4, CDH13, PKHD1, SLC22A12, MYO1C, USH1C, CYFIP1, NME2, ACTN4, EFHC1, SOD1, ITPR3, DFNB31, CDH23, TGFB2, CACNA1A, TMPRSS15, APBB2, MYO3A, PKD2, ITLN1, PDPN, FGD5, CLIC1, TLN1, PCDH15, FGD2, MYO1A, ACTN1, B4GALT1, DOCK4, CLIC5, IQCB1, GHSR, MYO6, DNAH9, NF1, DNAI1, RPGRIP1L, DNALI1, BCAR1, ARAP3, ITGB1, SPAG6, GAS8, ATP6V0A4, CCDC88A, LAYN, ENPP7, CLIC4, GHRL, MARK4, MTMR14, NEFH, S100B, CDK6, SI, EZR, DTNBP1, S100A6, NRSN1, FGD6, ALS2, CABP4, CD2AP, TSC1, CEP290, CDC42, OXCT2, CDK5, DNAI2, ARFIP2, RAB35, MYO7A, SYNPO, NEFM, CDK5R1, ROPN1B, PPT1, CLN3, MYO5A, ABI3, APBB1, DBNL, S100A11, MYH9, EVL, INPP5J, NEFL, INPP5K, ACTN3, ITGB1BP1, CTTN, ITGA5, ESPN, SCYL3, FAP, WASF2, ARF6, CROCC, (AKAP4), (FGD1), (AMOT)
MICROVILLUS	11	11	4.70E-06	TBC1D10A, MYO1C, USH1C, DFNB31, CDH23, PCDH15, MYO1A, DOCK4, CLIC5, ENPP7, CLIC4
NEURITE DEVELOPMENT	51	53	1.24E-05	RTN4, NRP1, APOE, MAPT, SLIT1, LRRC4C, NRTN, FEZ2, PARD6B, NRXN1, CYFIP1, UNC5C, CNTN4, ROBO1, ROBO2, PAX2, NRXN3, NTNG2, LST1, FEZ1, SEMA3B, TRAPPC4, BAIAP2, MAP1S, SPON2, PARD3, GLI2, YWHAH, LAMB1, GHRL, S100B, BAI1, DPYSL5, NTNG1, AMIGO1, SLIT2, ALS2, RTN4RL1, CDK5, GDNF, CDK5R1, NRP2, SIAH1, KLK8, POU4F1, SHH, OTX2, RTN4RL2, SEMA4F, UBB, THY1, (OPHN1), (KAL1)
CELL	18	19	1.31E-05	COLEC12, NTM, SPAM1, MICB, VCAN, OPCML, CNTN4,

RECOGNITION				CRTAM, CLEC7A, CADM1, CLEC4M, (CD209), MICA, AMIGO1, SLIT2, ZP2, CDK5R1, PCDH12, PECAM1
GENERATION OF NEURONS	80	83	1.42E-05	MDGA2, RTN4, NRP1, APOE, MAPT, SLIT1, LRRC4C, NRTN, FEZ2, PARD6B, NRXN1, CYFIP1, TGFB2, LMX1B, UNC5C, CNTN4, PCSK9, ROBO1, ROBO2, PAX2, NLGN1, NRXN3, NTNG2, FARP2, LST1, FEZ1, SEMA3B, ATP2B2, TRAPPC4, BTG4, BAIAP2, NF1, MAP1S, SPON2, LDB1, PARD3, GLI2, YWHAH, CIT, POU6F2, RND1, LAMB1, KCNIP2, GHRL, YWHAG, S100B, BAI1, DPYSL5, NTNG1, AMIGO1, SLIT2, ALS2, RTN4RL1, MDGA1, KRT2, PICK1, RTN1, CDK5, BRSK2, NPTN, GDNF, VWC2, CDK5R1, NRP2, DTX1, PPT1, RACGAP1, SIAH1, AGRN, NRCAM, SERPINF1, KLK8, POU4F1, SHH, OTX2, RTN4RL2, ARTN, SEMA4F, UBB, THY1, (OPHN1), (SMARCA1), (KAL1)
TRANSMEMBRANE RECEPTOR PROTEIN KINASE ACTIVITY	50	51	1.82E-05	NRP1, ACVR1, ERBB4, EPHA5, CRIM1, ROR1, ALK, ACVR1B, PDGFRB, (CSF1R), MET, FGFR4, EGFR, INSR, TEK, AXL, IGF1R, EPHB2, PDGFRL, RYK, IGF2R, NTRK1, FLT3, TRIM27, DDR2, FGFR1, MUSK, DDR1, ACVR2B, ACVRL1, TGFBR2, EFNA3, ERBB3, ROR2, ACVR1C, FLT1, EFNB3, MERTK, TIE1, KDR, FLT4, PTK7, NRP2, TYRO3, TGFBR1, MST1R, EPHB6, ACVR2A, PDGFRA, EFNA4, LTK
PROTEIN DOMAIN SPECIFIC BINDING	71	72	1.94E-05	TBC1D10A, BAX, WBP2NL, HOXB1, MAPT, ESRRG, CRK, USH2A, GRM7, PTEN, SLC22A12, NOD2, ADAM9, NLRP2, ELMO1, INSR, CARD11, PTPN12, LDB2, PAG1, TLN1, ARHGAP27, CCDC88C, PTK2, CADM1, DOCK4, BCL2, PPARGC1B, LDB1, SRC, HSP90AB1, ARHGEF4, YWHAH, KIF20B, MLF1, CARD8, NOS1AP, RAD9A, MICAL1, SQSTM1, ABI2, JAK2, CARD14, LAX1, CLCN3, NUP62, ARHGAP5, AFAP1L2, CD2AP, GPX1, CD3E, CARD9, SSTR2, USHBP1, SIT1, CFTR, LPAR2, ADAM15, EVL, PYCARD, RIPK2, FOXH1, STUB1, ERCC1, XPA, IRS1, ESPN, LCK, HSP90AA1, PDZK1, LAT2, (BPY2)
NEURON DEVELOPMENT	59	61	2.58E-05	RTN4, NRP1, APOE, MAPT, SLIT1, LRRC4C, NRTN, FEZ2, PARD6B, NRXN1, CYFIP1, TGFB2, UNC5C, CNTN4, ROBO1, ROBO2, PAX2, NRXN3, NTNG2, FARP2, LST1, FEZ1, SEMA3B, TRAPPC4, BAIAP2, MAP1S, SPON2, PARD3, GLI2, YWHAH, RND1, LAMB1, KCNIP2, GHRL, S100B, BAI1, DPYSL5, NTNG1, AMIGO1, SLIT2, ALS2, RTN4RL1, PICK1, CDK5, GDNF, CDK5R1, NRP2, PPT1, SIAH1, AGRN, NRCAM, KLK8, POU4F1, SHH, OTX2, RTN4RL2, SEMA4F, UBB, THY1, (OPHN1), (KAL1)
AXONOGENESIS	41	43	2.73E-05	RTN4, NRP1, APOE, MAPT, SLIT1, LRRC4C, FEZ2, PARD6B, NRXN1, CYFIP1, UNC5C, CNTN4, ROBO1, ROBO2, PAX2, NRXN3, NTNG2, FEZ1, SEMA3B, BAIAP2, SPON2, PARD3, GLI2, S100B, BAI1, DPYSL5, NTNG1, AMIGO1, SLIT2, RTN4RL1, CDK5R1, NRP2, SIAH1, KLK8, POU4F1, SHH, OTX2, RTN4RL2, SEMA4F, UBB, THY1, (KAL1), (OPHN1)
CELLULAR	47	49	4.07E-05	RTN4, NRP1, APOE, MAPT, SLIT1, LRRC4C, FEZ2, RORB,

MORPHOGENESIS DURING DIFFERENTIATION				PARD6B, NRXN1, CYFIP1, UNC5C, CNTN4, ROBO1, ROBO2, PAX2, NRXN3, NTNG2, FEZ1, SEMA3B, BAIAP2, MAP1S, SPON2, PARD3, GLI2, YWHAH, S100B, BAI1, DPYSL5, NTNG1, AMIGO1, SLIT2, ALS2, RTN4RL1, CEP290, CDK5R1, NRP2, SIAH1, KLK8, POU4F1, SHH, OTX2, RTN4RL2, SEMA4F, NRL, UBB, THY1, (OPHN1), (KAL1)
NEUROGENESIS	90	93	5.83E-05	MDGA2, RTN4, NRP1, APOE, MAPT, SLIT1, LRRC4C, NRTN, FEZ2, PARD6B, NRXN1, CYFIP1, SOD1, TGFB2, LMX1B, UNC5C, CNTN4, PCSK9, ROBO1, ROBO2, PAX2, NLGN1, NRXN3, EIF2B3, NTNG2, FARP2, LST1, FEZ1, SEMA3B, ATP2B2, TRAPPC4, BTG4, BAIAP2, NF1, MAP1S, SPON2, LDB1, PARD3, GLI2, YWHAH, CIT, POU6F2, RND1, LAMB1, KCNIP2, GHRL, YWHAG, CDK6, S100B, BAI1, DPYSL5, NTNG1, AMIGO1, SLIT2, ALS2, RTN4RL1, EIF2B5, MDGA1, AZU1, KRT2, NF2, PICK1, RTN1, CDK5, BRSK2, NPTN, GDNF, VWC2, EIF2B1, CDK5R1, NRP2, DTX1, PPT1, RACGAP1, SIAH1, AGRN, NRCAM, EIF2B4, SERPINF1, CLN5, KLK8, POU4F1, SHH, OTX2, RTN4RL2, EIF2B2, ARTN, SEMA4F, UBB, THY1, (OPHN1), (SMARCA1), (KAL1)
TRANSMEMBRANE RECEPTOR PROTEIN TYROSINE KINASE ACTIVITY	42	43	6.56E-05	NRP1, ERBB4, EPHA5, CRIM1, ROR1, ALK, CSF1R, (PDGFRB), MET, FGFR4, EGFR, INSR, TEK, AXL, IGF1R, EPHB2, PDGFRL, RYK, IGF2R, NTRK1, FLT3, TRIM27, DDR2, FGFR1, MUSK, DDR1, EFNA3, ERBB3, ROR2, FLT1, EFNB3, MERTK, TIE1, KDR, FLT4, PTK7, NRP2, TYRO3, MST1R, EPHB6, PDGFRA, EFNA4, LTK
VESICLE MEDIATED TRANSPORT	188	194	1.41E-04	CPNE1, RAB13, RPH3AL, COLEC12, STX18, ERGIC3, LRP1B, VAMP3, VPS33B, MAPK8IP3, TSC2, CDH13, NECAP2, SORL1, STEAP2, CADPS, SPACA3, ELMO1, CORO1C, EPN1, DOPEY2, ARFGEF1, ARFGEF2, ABCA1, STAB2, RAB7A, SEC23A, SCAMP1, PDLIM7, RAB14, LRP2, SCRN1, NLGN1, FCN2, LMAN2L, ARHGAP27, VTI1A, ATP6V1H, DOCK1, CLEC7A, RABEP1, FCN1, LIN7A, SNX2, SNAP23, SEC22A, SEC24B, AHSG, NAPA, PACSIN3, LRPAP1, GULP1, CPNE3, COPG2, SYT1, ZFYVE16, MYO6, PICALM, PLIN3, CYTH2, VPS4B, HIP1, IGF2R, GATA2, CPLX1, MSR1, KRT18, ERGIC2, RER1, COPE, LMAN1, ARAP3, RAB22A, SCIN, LDLRAP1, LRMP, COPB2, STX5, CLTCL1, SFTPD, GOSR1, ADORA2A, KALRN, SNX1, STX7, CYTH1, KIF20A, RAC1, KIF1C, SH3BP4, GOLGA5, TMX1, COG2, ANKRD27, SCAMP3, SNAP29, SQSTM1, AMPH, COG7, FXN, SPTBN2, EEA1, TOM1, LRP8, SYNJ1, ERGIC1, CHMP1A, STAB1, ITSN1, SYTL2, ACTR1A, PRKCI, MON2, STON1, CPLX2, AP1M2, AZU1, RAB26, RIMS1, M6PR, SPTBN4, LDLR, COG3, OPTN, EXOC5, CCL3, ADORA1, RAMP2, AP3B2, NAPG, RAB2A, RAB35, CBL, FOLR1, BET1, TINAGL1, NRBP1, CCL8, USE1, PPT1, SEC23B, CLN3, MAPK8IP1, LYST, GBF1, VPS4A, YKT6, DOPEY1, RAB5A, PKDREJ, CD14, ZW10, SNX3, CYTH3, RABEPK, COPB1, GOSR2, TMED10, GOLGA4, STX6, AP1S1, DNM1, STON2,

				RAMP3
GLUTAMATE RECEPTOR ACTIVITY	20	20	1.47E-04	GRM1, GRM6, GRM7, GRIN2A, GABBR2, GRIK1, GRM8, GRIN2B, GRIK2, GRIK4, GRIA1, GRM4, GRM2, GRIK3, GRM3, GRM5, GABBR1, GRIA2, GRIN2C, GRIK5
CYTOSKELETAL PROTEIN BINDING	153	159	1.59E-04	RPH3AL, SVIL, ABLIM1, DST, APOE, ACTN2, BRCA2, JUP, MAPT, ABI1, MAPK8IP3, SPTBN1, SORBS2, RAB11FIP5, NRAP, CYFIP1, ACTN4, IQGAP2, MYBPC1, SYNE1, DYNC1I1, WIPF1, RAE1, MYO16, PKD2, ARFGEF1, EGFR, FXYD5, ANLN, KATNA1, TNNC1, ARPC4, TLN1, GC, SHROOM3, MYO9B, DLG1, BIRC5, TMOD3, KPTN, ACTN1, PACSIN3, CALD1, ANK1, TUBGCP5, BAIAP2, MYO6, CENPJ, ADD2, MAP1S, TPPP, MTSS1, TMOD2, APC, CAPZB, ATG4C, MARCKS, SPTB, PALLD, ARL8A, NCALD, SCIN, SORBS3, CLASP1, PXN, KIF1B, PDLIM5, TRIM63, SPTA1, SPTAN1, MEFV, CCDC88A, TUBGCP3, PRNP, MARK4, SORBS1, NDE1, S100B, COTL1, LSP1, ABI2, SPTBN2, EZR, HPCA, ACTA1, ABRA, SSH2, SSH1, HOOK3, LRPPRC, TTN, CLASP2, SUN2, MAPK8IP2, KIF5B, TWF2, RABGAP1, CEP290, ATG4D, ARPC1A, VCL, MAPRE1, HTT, SPTBN4, CLIP1, PLS1, STMN1, TNNT2, FLII, ADD1, CDC42EP3, SYNPO, POLB, PARVG, GABARAPL2, RHCG, CNN2, MYLIP, MAPK8IP1, RACGAP1, PKD2L1, GABARAP, NEXN, NRCAM, TNNT1, CGN, TMOD4, DBNL, CDK5RAP2, LIMA1, CTNNA1, MYH9, RHAG, TARDBP, MYBPC3, SMTN, SHROOM1, MYH10, CAPZA1, TUBGCP6, TNNI3, CXCR4, ARL8B, MYOZ1, VAPB, ESPN, CCR5, ANG, IPP, WASF2, GABARAPL1, ARHGEF2, CROCC, (UXT), (DMD), (FLNA), (ATG4A), (SHROOM2), (DCX)
JNK CASCADE	45	47	1.70E-04	MDFIC, MAPK10, TPD52L1, DUSP10, MAPK8IP3, DAXX, SH2D3A, TNIK, MAP4K1, PTPLAD1, MAPK9, MAP3K12, GPS1, HIPK2, MINK1, MAP3K9, MAP4K2, MAP3K11, SH2D3C, MAP4K5, CD27, ZAK, MAPK8, GPS2, MAP3K13, MAP4K3, MAP2K4, MAP3K4, KIAA1804, MAP3K10, MAP3K5, TAOK2, MAPK8IP2, MDFI, CARD9, MAP3K2, CRKL, ZNF675, ADORA2B, MAPK8IP1, TAOK3, PKN1, DBNL, AMBP, MAP3K6, (EDA2R), (DUSP9)
STRESS ACTIVATED PROTEIN KINASE SIGNALING PATHWAY	47	49	1.96E-04	MDFIC, MAPK10, TPD52L1, DUSP10, MAPK8IP3, DAXX, SH2D3A, TNIK, MAP4K1, PTPLAD1, MAPK9, MAP3K12, GPS1, HIPK2, MINK1, MAP3K9, MAP4K2, MAP3K11, SH2D3C, MAP4K5, CD27, ZAK, MAPK8, GPS2, MAP2K7, MAP3K13, MAP4K3, CCM2, MAP2K4, MAP3K4, KIAA1804, MAP3K10, MAP3K5, TAOK2, MAPK8IP2, MDFI, CARD9, MAP3K2, CRKL, ZNF675, ADORA2B, MAPK8IP1, TAOK3, PKN1, DBNL, AMBP, MAP3K6, (EDA2R), (DUSP9)
METABOTROPIC GLUTAMATEGABAB LIKE RECEPTOR ACTIVITY	10	10	2.54E-04	GRM1, GRM6, GRM7, GABBR2, GRM8, GRM4, GRM2, GRM3, GRM5, GABBR1

PHAGOCYTOSIS	16	17	3.05E-04	COLEC12, SPACA3, ELMO1, CORO1C, FCN2, DOCK1, CLEC7A, FCN1, AHSG, GULP1, GATA2, SFTPD, ADORA2A, AZU1, ADORA1, CD14, (FCGR1A)
REGULATION OF AXONOGENESIS	10	10	3.15E-04	RTN4, APOE, MAPT, LRRC4C, ROBO1, ROBO2, AMIGO1, SLIT2, KLK8, THY1
REGULATION OF ANATOMICAL STRUCTURE MORPHOGENESIS	24	25	3.17E-04	RTN4, CDC42EP1, APOE, MAPT, LRRC4C, FGD4, ROBO1, ROBO2, FGD5, FGD2, CDC42EP4, YWHAH, ARAP3, AMIGO1, SLIT2, FGD6, TAOK2, CDC42, CDC42EP2, CDC42EP5, KLK8, ARAP1, MYH9, THY1, (FGD1)
PERINUCLEAR REGION OF CYTOPLASM	51	54	3.57E-04	WBP2NL, TPD52L1, HSP90B1, FAF1, YWHAB, CYFIP1, ACTN4, DYNC1I1, CAV2, MYO16, HMGB2, RAB14, MYO9B, ZFYVE1, TAF10, TPD52, CHODL, PARK2, MYO6, MAP1S, TPPP, CYFIP2, TYR, APEX1, DCTN3, NPC1, ATXN2, LAMB1, SLC2A4, TLR4, ATXN10, MTMR14, VAPA, LRPPRC, KIF5B, NF2, ANP32A, PICK1, BCL10, SPINK5, DNAJB6, CDH1, VPS4A, ACHE, TNFSF12, STX6, CLN5, SET, ATP7B, GTPBP4, AGGF1, (MAGEE1), (ATP7A)
GLUTAMATE SIGNALING PATHWAY	16	17	4.22E-04	GRID2, GRM6, GRIN2A, HOMER2, GRIK1, GRIN2B, GRIK2, GRIK4, GRIA4, HOMER3, GRM4, GRIK3, GRM5, HOMER1, CDK5R1, GRIN2C, (GRIA3)
CORNIFIED ENVELOPE	12	13	4.79E-04	SPRR1B, DSP, CDSN, CSTA, SPRR1A, (SPRR3), IVL, LOR, ANXA1, SCEL, EVPL, CNFN, TGM1
LIPOPROTEIN BINDING	18	18	5.27E-04	LRP1, COLEC12, MAPT, CDH13, SORL1, STAB2, CXCL16, APOA4, LRPAP1, LRP6, TLR6, STAB1, ANKRA2, LDLR, APOBR, VLDLR, APOL2, SCARF1
PDZ DOMAIN BINDING	14	14	5.64E-04	TBC1D10A, USH2A, GRM7, PTEN, SLC22A12, CCDC88C, CADM1, DOCK4, NOS1AP, CLCN3, SSTR2, USHBP1, CFTR, PDZK1
PROTEIN TYROSINE KINASE ACTIVITY	62	63	6.21E-04	NRP1, ERBB4, EPHA5, CRIM1, ROR1, TNK2, ALK, PDGFRB, (CSF1R), MET, LYN, FGFR4, EGFR, INSR, TEK, AXL, IGF1R, EPHB2, PDGFRL, TWF1, RYK, CLK1, IGF2R, NTRK1, FLT3, TRIM27, DDR2, KIT, FGFR1, MUSK, DDR1, SKAP1, EFNA3, TXK, PTK2B, ERBB3, ITK, FRK, ROR2, FLT1, CAMKK2, EFNB3, MERTK, TIE1, KDR, DYRK1A, FLT4, CCL4, TTK, PTK7, PTK6, CRKL, DYRK2, NRP2, TYRO3, TYK2, MST1R, EPHB6, ERBB2, PDGFRA, EFNA4, NR4A3, LTK
3 5 CYCLIC NUCLEOTIDE PHOSPHODIESTERASE ACTIVITY	13	13	6.39E-04	PDE3A, PDE4B, PDE1C, PDE4D, PDE10A, PDE1B, PDE7A, PDE1A, PDE3B, PDE2A, PDE7B, PDE4C, PDE4A
NEGATIVE REGULATION OF CELL PROLIFERATION	148	156	7.07E-04	OSM, PDS5B, UMOD, NCK2, BRCA2, TOB2, ABI1, DLG5, ATP8A2, CDH13, FTH1, PTEN, COL18A1, GNRH1, RBBP4, NME1, NME2, UTP20, RUNX3, TNFSF15, OPRM1, TGFB2, MYO16, VHL, MNT, CDKN2A, MXI1, IL1B, MFN2, ING1, CHEK1, PTHLH, RARRES1, SSTR1, ADRA1A, TBX5, IGFBP7, LST1, EREG, GPNMB, TOB1, KRT4, CDKN1C, CDKN2B, QSOX1, AIF1, BTG4, NF1, APC, CCL23, CTBP1, EEF1E1,

				CDKN2C, COL4A3, SCIN, BTG2, SFTPD, MDM2, ETS1, ALOX15B, CD33, GAS8, TNFRSF9, TBRG1, ACVRL1, KAT2B, ENPP7, GHRL, POU1F1, TNFRSF8, CDK6, BAI1, IGFBP6, BTG3, FRK, SSTR5, NOTCH2, PLG, WARS, AIMP1, TGFB1I1, EIF2AK2, NUP62, RERG, CD164, SSTR3, NPM1, TSC1, CNOT8, CTBP2, IL6, ADAMTS1, HGS, CDKN2D, ATPIF1, NF2, CXCL1, NOX4, SSTR2, CDKN3, FGFBP1, SST, SIRPG, FABP6, TM4SF4, BAP1, MDM4, BNIPL, KLF11, SESN1, DLEC1, NDN, CUL2, PRKRIR, ADAMTS8, KLF10, BTG1, IFITM1, EMP3, SSTR4, PAWR, CDK10, SCG2, KLF4, TP53I11, S100A11, FABP7, ING4, IL8, GML, CGREF1, CDC6, FABP3, CUL5, CUL4A, GLMN, GTPBP4, SPEG, B4GALT7, CUL1, ANG, IL1A, MXD4, CGRRF1, RARRES3, PRKRA, ING5, PPM1D, (FOXO4), (MAGED1), (ODZ1), (IL29), (FOXP3), (LDOC1), (CCL3L3)
PROTEIN OLIGOMERIZATIO N	35	40	7.26E-04	BAX, APOE, ACTN2, CDA, NOD2, EIF2AK3, AMFR, INSR, IGF1R, CCDC88C, GOPC, TP63, SYT1, VWF, MAP3K11, SCUBE1, MALT1, STOM, AASS, AKR1C1, NOD1, SCUBE3, NLRP3, TGM3, TRPV5, ATPIF1, GPX3, BCL10, ALDH5A1, DGKD, UPK1A, TP53, RAD51, MUC20, PEX14, (HPRT1), (IRAK1)
EXOPEPTIDASE ACTIVITY	29	32	7.45E-04	DPP4, TRHDE, GGH, ERAP1, TPP1, METAP1D, PEPD, XPNPEP1, LNPEP, ANPEP, CPD, ERAP2, ENPEP, BLMH, ACE, ZMPSTE24, DPEP1, AEBP1, CPB2, METAP2, BACE1, C9orf3, METAP1, CPB1, CPE, CPA2, (CPA3), UCHL1, CPA1, NPEPPS, (ACE2), (PGCP)
EXTRACELLULAR MATRIX	95	100	8.17E-04	MEPE, LAMA2, DMP1, FMOD, DST, SGCB, DSPP, FBLN5, VCAN, EFEMP1, USH2A, SNTB1, COL18A1, SOD1, THBS4, SMC3, LAMA4, COL8A1, COL15A1, ECM1, SNTG2, COL5A1, COL9A3, MATN3, IMPG2, CHAD, COL1A2, FBLN1, COL4A2, COL13A1, LTBP2, COL9A2, COL4A3, AMTN, SSPN, SGCD, FBN2, HAPLN1, SPG7, SNTG1, APLP1, EFEMP2, LAMB1, TNXB, COL9A1, COL10A1, ERBB2IP, COL6A3, TINAG, CAV3, MMP10, COL16A1, TFPI2, COL7A1, ADAMTS5, FBLN2, ADAMTS9, POSTN, SNTB2, SGCE, MGP, CHI3L1, CTGF, COL11A1, LTBP4, NID2, SGCA, SGCG, ODAM, COLQ, ADAMTS13, IMPG1, AGRN, MATN1, COL5A2, COL3A1, LAMB2, DGCR6, MUC2, COL4A4, COL5A3, LAMA3, ECM2, CD248, LAMC1, TGFB1, LUM, PI3, FBN1, COMP, PRSS2, ANG, MMP11, OPTC, PRELP, (MAGEE1), (DMD), (KAL1), (MUC5AC), (COL4A5)
CELL CELL ADHESION	83	86	8.28E-04	PVRL2, SYK, CDH13, PKHD1, PTEN, CADM3, CDSN, LGALS7, CLDN22, NCAM2, CDH5, CNTN4, ROBO1, EGFR, ROBO2, TNF, CDKN2A, VANGL2, NLGN1, DLG1, REG3A, CADM1, APOA4, BMP1, CLDN20, CLDN4, CD209, CD47, RASA1, COL13A1, CYFIP2, EMCN, CLDN3, B4GALNT2, ITGB1, CD84, CTNNA3, CELSR1, CLDN14, CLDN10, ACVRL1, AMIGO2, CD93, ITGB2, CLDN19, CLDN12, CLDN17, PVRL3, LMO4, AMIGO1, PKD1, CD164, NINJ2, CLDN18, PVRL1, AMIGO3,

				ATP2C1, CLDN9, (CLDN6), NF2, CLDN1, MGP, CERCAM, COL11A1, SIRPG, NPTN, CRNN, BCL10, CLDN11, CDK5R1, CLDN5, CD34, CX3CL1, CLDN7, CLDN23, ALX1, ANXA9, CLDN16, MPZL2, GTPBP4, CLDN15, CLDN8, CALCA, THY1, (CLDN2), (TRO)
PROTEINACEOUS EXTRACELLULAR MATRIX	93	98	8.47E-04	MEPE, LAMA2, DMP1, FMOD, DST, SGCB, DSPP, FBLN5, VCAN, EFEMP1, USH2A, SNTB1, COL18A1, THBS4, SMC3, LAMA4, COL8A1, COL15A1, ECM1, SNTG2, COL5A1, COL9A3, MATN3, IMPG2, CHAD, COL1A2, FBLN1, COL4A2, COL13A1, LTBP2, COL9A2, COL4A3, AMTN, SSPN, SGCD, FBN2, HAPLN1, SPG7, SNTG1, APLP1, EFEMP2, LAMB1, TNXB, COL9A1, COL10A1, ERBB2IP, COL6A3, TINAG, CAV3, MMP10, COL16A1, TFPI2, COL7A1, ADAMTS5, FBLN2, ADAMTS9, POSTN, SNTB2, SGCE, MGP, CHI3L1, CTGF, COL11A1, LTBP4, NID2, SGCA, SGCG, ODAM, COLQ, ADAMTS13, IMPG1, AGRN, MATN1, COL5A2, COL3A1, LAMB2, DGCR6, MUC2, COL4A4, COL5A3, LAMA3, ECM2, CD248, LAMC1, TGFB1, LUM, PI3, FBN1, COMP, ANG, MMP11, OPTC, PRELP, (MAGEE1), (KAL1), (DMD), (MUC5AC), (COL4A5)
MAINTENANCE OF PROTEIN LOCALIZATION	12	13	8.50E-04	TAF3, FAF1, TOPORS, NFKBIL1, MXI1, PDIA2, NFKBIE, SRGN, TONSL, MDFI, SUPT7L, PDIA3, (TMSB4Y)
MAINTENANCE OF CELLULAR PROTEIN LOCALIZATION	11	11	9.02E-04	TAF3, FAF1, TOPORS, NFKBIL1, MXI1, PDIA2, NFKBIE, TONSL, MDFI, SUPT7L, PDIA3
TRANSMEMBRANE RECEPTOR PROTEIN PHOSPHATASE ACTIVITY	19	19	9.21E-04	PTPRT, PTPRD, PTPRG, PTPRN2, PTPRO, PTPRM, PTPRS, PTPRB, PTPRZ1, PTPRH, PTPRK, PTPRC, PTPRR, PTPRA, PTPRU, PTPRE, PTPRN, PTPRF, PTPRJ
CELL PROJECTION BIOGENESIS	23	25	9.66E-04	CDC42EP1, ACTN2, FGD4, CDH13, CYFIP1, THBS4, FGD5, VANGL2, FGD2, CDC42EP4, RAC3, CCDC88A, RAC1, PCNT, FGD6, CD2AP, VCL, CDC42, DNAI2, ARFIP2, CDC42EP2, CDC42EP5, ARAP1, (FGD1), (OPHN1)
CYCLIC NUCLEOTIDE PHOSPHODIESTERASE ACTIVITY	14	14	0.001002979	PDE3A, PDE4B, PDE1C, PDE4D, PDE10A, PDE1B, PDE7A, PDE1A, PDE3B, PDE2A, CNP, PDE7B, PDE4C, PDE4A
CENTRAL NERVOUS SYSTEM DEVELOPMENT	110	123	0.001027387	MDGA2, DNER, SLIT3, CNTN6, SLIT1, NHLH2, PTEN, ADAM22, ALK, RNF103, FOXP2, JARID2, MYO16, EGR2, UNC5C, CNTN4, MBP, GRIK1, ROBO2, SH3GL3, SNTG2, NPAS2, ALDH3A2, ZBTB16, EIF2B3, NEUROG3, PSPN, RCAN1, PBX3, SH3GL2, DSCAML1, POU3F3, PROP1, PARK2, NKX2-2, NF1, MAP1S, BPTF, NCOA6, GLI2, NNAT, PTPRZ1, SOX8, CELSR1, POU6F2, PAX6, ADORA2A, PBX1, DMBX1,

				PHGDH, PCP4, DCLK1, HPCAL4, GPR56, MOG, BTD, S100B, ADAM23, DLX2, PCDH18, MAL, NPTX1, ARNT2, FOXG1, PDGFC, SHC3, TBR1, ZIC1, PITPNM1, EIF2B5, CEP290, MDGA1, POU6F1, SIX3, NPAS1, PNMA1, SERPINI1, ATN1, SH3GL1, PPP1R17, NDUFS4, ECE2, CTNS, EIF2B1, CDK5R1, UBE3A, PPT1, ALDH5A1, GSTP1, JRKL, SNCA, PBX4, EIF2B4, ALX1, LHX6, NCKAP1, CLN5, TAGLN3, SHH, OTX2, B3GNT5, ZIC2, UGT8, NMUR2, NHLH1, EIF2B2, WNT1, HESX1, UTP3, PTS, (SMARCA1), (SOX3), (MYT2), (IL1RAPL2), (VCX3A), (SHROOM2), (SHROOM4), (DCX), (AFF2), (ACCN1), (RPS6KA6), (RPS6KA3), (DRP2)
PROTEIN TYROSINE PHOSPHATASE ACTIVITY	52	53	0.001074914	PTPRT, PTPRD, PTPN22, PTEN, PTPN6, PTPRG, EPM2A, PTPRN2, PTPRO, PTPRM, PTP4A3, PTPN5, PTPN3, PTPN12, DUSP2, DUSP1, PTPRS, MTMR3, PTPN1, PTPN7, PTPRB, CDC14B, PTPRZ1, PTPRH, PTPN14, PTPN11, PTPRK, PTPN21, DUSP12, PTPRC, DUSP6, PTPRR, PTPRA, PTP4A2, MTMR7, TIMM50, PTPRU, DUSP5, PTPRE, DUSP8, PTPRN, CDC25B, PTPN2, PTPN4, PTPRF, PTPN9, PTPLA, DUSP3, PTPRJ, DUSP11, PTPN18, DUSP7, (TPTE)
ACTIVE TRANSMEMBRANE TRANSPORTER ACTIVITY	113	122	0.001433007	SLC7A2, SLC5A7, ATP1B1, FXYD2, SLC16A7, SLC38A3, SLC6A6, SLC12A4, SLC20A1, SLC7A4, TAPBP, ABCB11, SLC4A4, SLC17A5, SLC12A3, AQP9, ATP6V1C1, ABCD3, SLC5A1, PDPN, SLC9A2, SLC1A5, SLC17A7, ABCG2, SLC22A11, ABCF1, SLC6A3, SLC22A3, ATP1A2, SLC1A2, OCA2, SERINC1, SLC6A4, SLC9A5, ATP8B1, ABCC1, ABCC6, SLC6A5, ATP6V0E1, SLC13A2, SLC9A3, SLC43A1, ABCC3, SLC6A2, SLC20A2, SLC25A12, SLC44A2, ANKH, SLC25A15, SLCO1B1, ATP1B3, SLC1A6, ABCG1, SLC1A1, SLC18A3, SLC3A1, SLC1A4, SLC5A3, SLC25A11, SLC15A1, ATP2B4, SLC12A2, SLC5A2, SLC15A2, ATP1A1, SLC25A22, ABCC2, ATP6V1B2, ATP11B, SLC10A2, ATP1A4, ATP1B2, SLC22A4, ATP2A3, SLC5A6, ATP1A3, SLC6A9, SLC22A7, ATP2C1, ATP2A2, SLC12A7, ABCA3, SLC7A9, SLC13A4, ATP4B, SLC12A1, (SLC18A1), ABCA8, SLC1A3, CTNS, SLC7A6, SLC18A2, SLC7A11, SLC1A7, SLC7A8, SLC12A9, SLC3A2, SLC25A13, SLC17A2, ATP2B1, SLC17A4, SLC10A1, SLC9A1, SLC7A5, SLC7A10, ATP4A, ATP7B, SLC17A3, SLC4A3, SLC34A3, SLC44A1, SEC61B, ABCD4, ATP2A1, (SLC9A7), (SLC9A6), (ATP2B3), (ATP6V0C), (SLC6A14), (ABCB7), (ATP7A), (SLC6A8)
NEURON DIFFERENTIATION	73	76	1.88E-06	MDGA2, RTN4, NRP1, APOE, MAPT, SLIT1, LRRC4C, NRTN, FEZ2, PARD6B, NRXN1, CYFIP1, TGFB2, LMX1B, UNC5C, CNTN4, PCSK9, ROBO1, ROBO2, PAX2, NLGN1, NRXN3, NTNG2, FARP2, LST1, FEZ1, SEMA3B, ATP2B2, TRAPPC4, BTG4, BAIAP2, MAP1S, SPON2, LDB1, PARD3, GLI2, YWHAH, POU6F2, RND1, LAMB1, KCNIP2, GHRL, YWHAG, S100B, BAI1, DPYSL5, NTNG1, AMIGO1, SLIT2, ALS2, RTN4RL1, MDGA1, PICK1, RTN1, CDK5, BRSK2, GDNF, VWC2, CDK5R1,

				NRP2, DTX1, PPT1, SIAH1, AGRN, NRCAM, KLK8, POU4F1, SHH, OTX2, RTN4RL2, SEMA4F, UBB, THY1, (OPHN1), (SMARCA1), (KAL1)
CELL PROJECTION	105	108	2.14E-06	TESC, TBC1D10A, ACTN2, MAPT, ABI1, FGD4, CDH13, PKHD1, SLC22A12, MYO1C, USH1C, CYFIP1, NME2, ACTN4, EFHC1, SOD1, ITPR3, DFNB31, CDH23, TGFB2, CACNA1A, TMPRSS15, APBB2, MYO3A, PKD2, ITLN1, PDPN, FGD5, CLIC1, TLN1, PCDH15, FGD2, MYO1A, ACTN1, B4GALT1, DOCK4, CLIC5, IQCB1, GHSR, MYO6, DNAH9, NF1, DNAI1, RPGRIP1L, DNALI1, BCAR1, ARAP3, ITGB1, SPAG6, GAS8, ATP6V0A4, CCDC88A, LAYN, ENPP7, CLIC4, GHRL, MARK4, MTMR14, NEFH, S100B, CDK6, SI, EZR, DTNBP1, S100A6, NRSN1, FGD6, ALS2, CABP4, CD2AP, TSC1, CEP290, CDC42, OXCT2, CDK5, DNAI2, ARFIP2, RAB35, MYO7A, SYNPO, NEFM, CDK5R1, ROPN1B, PPT1, CLN3, MYO5A, ABI3, APBB1, DBNL, S100A11, MYH9, EVL, INPP5J, NEFL, INPP5K, ACTN3, ITGB1BP1, CTTN, ITGA5, ESPN, SCYL3, FAP, WASF2, ARF6, CROCC, (AKAP4), (FGD1), (AMOT)
MICROVILLUS	11	11	4.70E-06	TBC1D10A, MYO1C, USH1C, DFNB31, CDH23, PCDH15, MYO1A, DOCK4, CLIC5, ENPP7, CLIC4

Table S2. Full list of Molecular Signatures Database (MSigDB) GO Ontology pathways enriched with hippocampal atrophy over 2 years from baseline.

set name	gene count	set size	p-value	corrected p-value	gene symbols
CELLULAR MORPHOGENESIS DURING DIFFERENTIATION	33	49	9.41E-06	0.008223803	LRRC4C, MAPT, NRXN3, NTNG1, RORB, PARD3, UNC5C, CYFIP1, MAP1S, CNTN4, ROBO2, SLIT1, PAX2, APOE, AMIGO1, SPON2, BAIAP2, NRP1, NRXN1, RTN4RL1, BAI1, NRP2, NRL, OTX2, GLI2, DPYSL5, ROBO1, SLIT2, S100B, SHH, NTNG2, CDK5R1, THY1, (ALS2), (RTN4), (CEP290), (POU4F1), (SEMA3B), (SEMA4F), (OPHN1), (SIAH1), (UBB), (KAL1), (RTN4RL2), (PARD6B), (KLK8), (YWHAH), (FEZ2), (FEZ1)
REGULATION OF ANATOMICAL STRUCTURE MORPHOGENESIS	18	25	1.12E-05	0.008223803	LRRC4C, ARAP3, MAPT, ARAP1, ROBO2, CDC42, APOE, CDC42EP5, AMIGO1, CDC42EP2, MYH9, FGD5, CDC42EP4, ROBO1, FGD4, SLIT2, FGD2, THY1, (RTN4), (FGD1), (CDC42EP1), (FGD6), (KLK8), (TAOK2), (YWHAH)
NEURITE DEVELOPMENT	34	53	1.76E-05	0.008223803	LRRC4C, MAPT, NRXN3, NTNG1, PARD3, UNC5C, CYFIP1, MAP1S, CNTN4, ROBO2, LST1, SLIT1, PAX2, APOE, AMIGO1, SPON2,

					BAIAP2, LAMB1, NRP1, NRXN1, RTN4RL1, BAI1, NRP2, OTX2, GLI2, GDNF, DPYSL5, ROBO1, SLIT2, S100B, SHH, NTNG2, CDK5R1, THY1, (RTN4), (ALS2), (NRTN), (POU4F1), (SEMA3B), (CDK5), (SEMA4F), (OPHN1), (GHRL), (TRAPPC4), (SIAH1), (UBB), (KAL1), (RTN4RL2), (PARD6B), (KLK8), (YWHAH), (FEZ2), (FEZ1)
AXONOGENESIS	30	43	5.95E-05	0.01301048	LRRC4C, MAPT, NRXN3, NTNG1, PARD3, UNC5C, CYFIP1, CNTN4, ROBO2, SLIT1, PAX2, APOE, AMIGO1, SPON2, BAIAP2, NRP1, NRXN1, RTN4RL1, BAI1, NRP2, OTX2, GLI2, DPYSL5, ROBO1, SLIT2, S100B, SHH, NTNG2, CDK5R1, THY1, (RTN4), (KAL1), (POU4F1), (RTN4RL2), (SEMA3B), (PARD6B), (KLK8), (SEMA4F), (OPHN1), (SIAH1), (UBB), (FEZ2), (FEZ1)
CELL CELL ADHESION	54	86	6.17E-05	0.01301048	CADM1, COL11A1, NCAM2, CTNNA3, PKHD1, BMP1, CD93, NLGN1, CDH13, PVRL2, CNTN4, ROBO2, CLDN22, TNF, SIRPG, CDSN, ITGB2, CLDN5, ANXA9, NINJ2, CLDN23, AMIGO1, DLG1, PVRL3, VANGL2, ACVRL1, COL13A1, CYFIP2, ITGB1, CLDN19, CADM3, CD209, CELSR1, CLDN7, NF2, BCL10, ROBO1, AMIGO2, LMO4, CLDN12, CX3CL1, CD47, PTEN, CLDN15, CLDN10, CLDN1, B4GALNT2, SYK, CLDN9, (CLDN6), CDK5R1, CLDN16, CLDN14, THY1, CD34, (CLDN8), (MPZL2), (CLDN4), (CLDN3), (APOA4), (REG3A), (CDKN2A), (ALX1), (EGFR), (GTPBP4), (MGP), (CD164), (CERCAM), (CD84), (AMIGO3), (ATP2C1), (NPTN), (CLDN2), (CLDN17), (CLDN18), (EMCN), (CRNN), (CLDN11), (CDH5), (CALCA), (PVRL1), (TRO), (PKD1), (RASA1), (LGALS7), (CLDN20)
NEURON DEVELOPMENT	40	61	3.44E-04	0.050177457	LRRC4C, MAPT, NRXN3, NTNG1, PARD3, UNC5C, CYFIP1, MAP1S, CNTN4, ROBO2, PPT1, LST1, SLIT1, PAX2, APOE, AMIGO1, SPON2, BAIAP2, LAMB1, NRP1, NRXN1, RTN4RL1, BAI1, NRP2, OTX2, GLI2, RND1, GDNF, DPYSL5, ROBO1, SLIT2, KCNIP2, S100B, PICK1, SHH, NTNG2, CDK5R1, NRCAM, TGFB2, THY1, (ALS2), (RTN4), (NRTN), (SEMA3B), (POU4F1), (CDK5), (FARP2), (SEMA4F), (TRAPPC4), (GHRL), (OPHN1), (SIAH1), (UBB), (KAL1),

					(RTN4RL2), (AGRN), (PARD6B), (KLK8), (YWHAH), (FEZ2), (FEZ1)
TRANSMEMBRANE RECEPTOR PROTEIN PHOSPHATASE ACTIVITY	15	19	3.45E-04	0.050177457	PTPRG, PTPRN2, PTPRT, PTPRD, PTPRR, PTPRB, PTPRC, PTPRM, PTPRK, PTPRA, PTPRS, PTPRE, PTPRU, PTPRO, PTPRJ, (PTPRF), (PTPRZ1), (PTPRH), (PTPRN)

Figure S1. The MDS plot for the ADNI non-Hispanic Caucasian participants

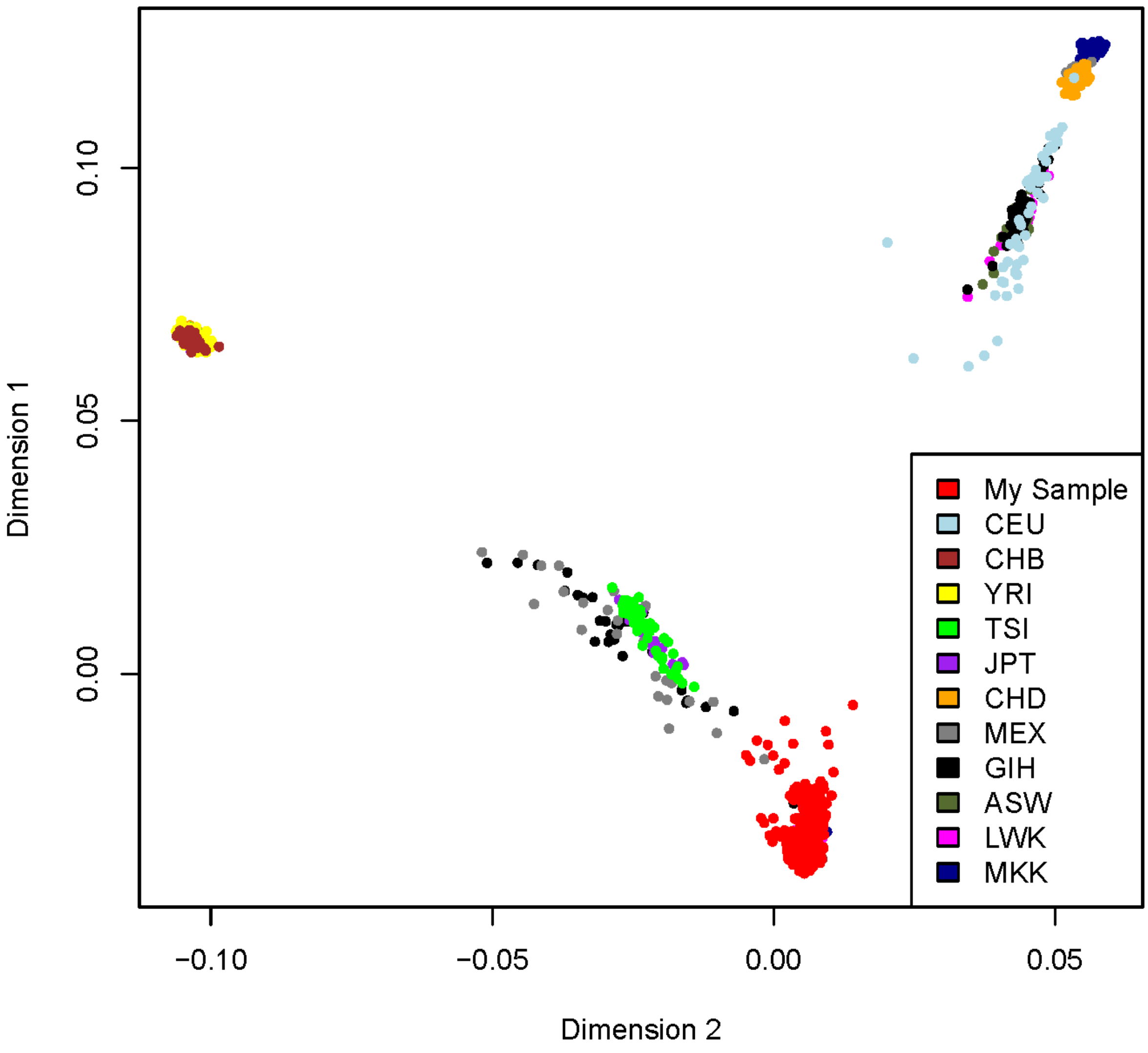

Supplement: Supplementary file 1 — Dataset 1 [file 41598_2019_50507_MOESM1_ESM.pdf]
